# Endocrine Disruption and Flame-Retardant Chemicals: PBDE-99 Effects on Rat Sexual Development

**Published:** 2006-02

**Authors:** Ernie Hood

Chemical flame retardants containing polybrominated diphenyl ethers (PBDEs) have attracted growing attention in recent years from toxicologists. Unlike their chemical cousins, dioxins and polychlorinated biphenyls (PCBs), environmental levels of PBDE compounds are increasing, as are concentrations found in human breast milk. This is of concern because of the potential that PBDEs may cause developmental toxicity due to endocrine-modulating effects, as has previously been documented with dioxins and PCBs. Results of a new German study examining developmental exposure in rats to a commonly used and environmentally abundant PBDE compound, PBDE-99, lend support to suspicions that PBDEs can have detrimental effects on sexual development in the offspring of exposed mothers **[*EHP* 114:194–201]**.

The researchers examined sexual development end points—circulating sexual steroid concentrations, reproductive organ development, and sexually dimorphic behavior—that have been shown in similar rodent experiments to change following exposure to a mixture of PCBs reconstituted according to the congener pattern found in human breast milk. In the current study, weight-matched groups of dams received daily subcutaneous injections of PBDE-99 from gestational days 10 through 18 at doses of 1 or 10 milligrams per kilogram body weight (mg/kg). For comparison, another group was dosed with 30 mg/kg of the PCB mixture Aroclor 1254 (A1254).

In PBDE-exposed male offspring, pronounced decreases in circulating sex steroids (estradiol, testosterone) were seen both at weaning and in adulthood. Anogenital distance, an androgen-dependent marker of sexual development, was also reduced in the male offspring. The males further displayed a dose-dependent increased preference for sweets, which is a sexually dimorphic behavior in rodents—this finding indicates feminization in the males. A slight acceleration in onset of puberty was noted in the low-dose group.

The PBDE-exposed females had less severe effects. Onset of puberty was mildly delayed in the high-dose group. Also, the number of primordial/primary (rudimentary) ovarian follicles was reduced in the low-dose group, with a more pronounced decline in secondary (more developed) follicles within the high-dose group. These results could indicate an impaired reproductive life span, in that ovarian follicles are a key indicator of ovarian health. The females also had an insignificant increase in sweet preference.

The researchers found no effect on sweet preference in male or female offspring exposed to the PCB mixture, but in the females they did see a significant increase in the number of tertiary (still larger, more developed) follicles. The exposure also adversely affected steroid concentrations in males and sexual development in both sexes. Thus, it appears that the pattern of endocrine-disruptive effects differs for PBDE-99 and A1254, which may be due to the higher content of dioxin-like compounds in the PCB mixture.

The scientists also examined organ weights as an indicator of overall development in the rodents. They found that pituitary gland weights were slightly decreased in males in the PBDE-99 high-dose group at weaning, while they were increased in females in the low-dose group. The authors speculate that these effects may indicate a biphasic response to PBDE-99, and suggest that the phenomenon should be studied further in future research. Results also included a marked dose-dependent decrease in thyroid gland weight in adult rats of both sexes exposed to either PBDE-99 or A1254.

This study adds to a growing body of literature indicating that chemical flame retardants are endocrine-disrupting compounds with the potential to profoundly affect sexual development and sexually dimorphic behaviors. The fact that several effects were seen in adulthood, long after termination of the exposure, indicates that the rats’ gestational exposure caused permanent physiologic damage. With increases in human body burden and environmental levels, and with the compounds’ apparent persistence in both venues, it is safe to assume that PBDEs will be the focus of more urgent research activity.

